# A new species of the Asian leaf litter toad genus *Leptobrachella* (Amphibia, Anura, Megophryidae) from southwest China

**DOI:** 10.3897/zookeys.943.51572

**Published:** 2020-06-22

**Authors:** Shi-Ze Li, Jing Liu, Gang Wei, Bin Wang

**Affiliations:** 1 Department of Food Science and Engineering, Moutai Institute, Renhuai 564500, China Moutai Institute Renhuai China; 2 CAS Key Laboratory of Mountain Ecological Restoration and Bioresource Utilization and Ecological Restoration Biodiversity Conservation Key Laboratory of Sichuan Province, Chengdu Institute of Biology, Chinese Academy of Sciences, Chengdu 610041, China Chengdu Institute of Biology, Chinese Academy of Sciences Chengdu China; 3 Biodiversity Conservation Key Laboratory, Guiyang College, Guiyang, 550002, China Guiyang College Guiyang China

**Keywords:** Guizhou, molecular phylogenetic analyses, morphology, new species, taxonomy

## Abstract

A new species of the Asian leaf litter toad genus *Leptobrachella* from Guizhou Province, China is described based on molecular phylogenetic analyses, morphological comparisons, and bioacoustics data. Phylogenetic analyses based on the mitochondrial 16S rRNA gene sequences supported the new species as an independent clade nested into the *Leptobrachella* clade and sister to *L. bijie*. The new species could be distinguished from its congeners by a combination of the following characters: small body size (SVL 30.8–33.4 mm in seven adult males, and 34.2 mm in one adult female); dorsal skin shagreened, some of the granules forming longitudinal short skin ridges; tympanum distinctly discernible, slightly concave; internasal distance longer than interorbital distance; supra-axillary, femoral, pectoral and ventrolateral glands distinctly visible; absence of webbing and lateral fringes on fingers; toes with rudimentary webbing and shallow lateral fringes, relative finger lengths II < IV < I < III; heels overlapped when thighs are positioned at right angles to the body; and tibia-tarsal articulation reaches the tympanum.

## Introduction

The Asian leaf litter toads of the genus *Leptobrachella* Smith, 1925 (Anura, Megophryidae) are widely distributed from southern China west to northeastern India and Myanmar, through mainland Indochina to peninsular Malaysia and the island of Borneo (Frost 2020). Many species in this genus had been ever classified into *Leptolalax* Dubois, 1983 (e.g., Fei et al. 2009, 2012), and Chen et al. (2018) placed *Leptolalax* as a junior synonym of *Leptobrachella* based on large-scale molecular analyses. Currently, the genus *Leptobrachella* contains 76 species, of which44 species have been described in the past ten years (Frost 2020). Currently, 21 species of the genus *Leptobrachella* are known from China: *Leptobrachella alpina* (Fei, Ye & Li, 1990) and *L. bourreti* (Dubois, 1983) from Yunnan and Guangxi; *L. eos* (Ohler, Wollenberg, Grosjean, Hendrix, Vences, Ziegler & Dubois, 2011) and *L. nyx* (Ohler, Wollenberg, Grosjean, Hendrix, Vences, Ziegler & Dubois, 2011) from Yunnan; *L. laui* (Sung, Yang & Wang, 2014) and *L. yunkaiensis* Wang, Li, Lyu & Wang, 2018 from southern Guangdong, including Hong Kong; *L. liui* (Fei & Ye, 1990) from Fujian, Jiangxi, Guangdong, Guangxi, Hunan, and Guizhou; *L. oshanensis* (Liu, 1950) from Gansu, Sichuan, Chongqing, Guizhou, and Hubei; *L. purpuraventra* Wang, Li, Li, Chen & Wang, 2019, *L. bijie* Wang, Li, Li, Chen & Wang, 2019, and *L. suiyangensis* Luo, Xiao, Gao & Zhou, 2020 from Guizhou; *L. purpura* (Yang, Zeng & Wang, 2018), *L. pelodytoides* (Boulenger, 1893), *L. tengchongense* (Yang, Wang, Chen & Rao, 2016), and *L. yingjiangensis* (Yang, Zeng & Wang, 2018) from Yunnan; *L. ventripunctata* (Fei, Ye & Li, 1990) from Guizhou and Yunnan; *L. mangshanensis* (Hou, Zhang, Hu, Li, Shi, Chen, Mo & Wang, 2018) from southern Hunan; and *L. sungi* (Lathrop, Murphy, Orlov & Ho, 1998), *L. maoershanensis* (Yuan, Sun, Chen, Rowley & Che, 2017), *L. shangsiensis* Chen, Liao, Zhou & Mo, 2019, and *L. wuhuangmontis* Wang, Yang & Wang, 2018 from Guangxi (Sung et al. 2014; Li et al. 2016; Yang et al. 2016, 2018; Yuan et al. 2017; Chen et al. 2018, 2019; Hou et al. 2018; Wang et al. 2018, 2019; Wang et al. 2019; Luo et al. 2020). Even more, a series of cryptic species in the genus were still proposed in Chen et al. (2018).

In recent years, we carried out a series of biodiversity surveys in Chishui City, Guizhou Province, China, and collected some specimens of the genus *Leptobrachella*. Molecular phylogenetic analyses, morphological comparisons, and bioacoustics comparisons consistently indicated these specimens as an undescribed species of *Leptobrachella*. Hence, we describe it herein as a new species.

## Materials and methods

**Figure 1.**
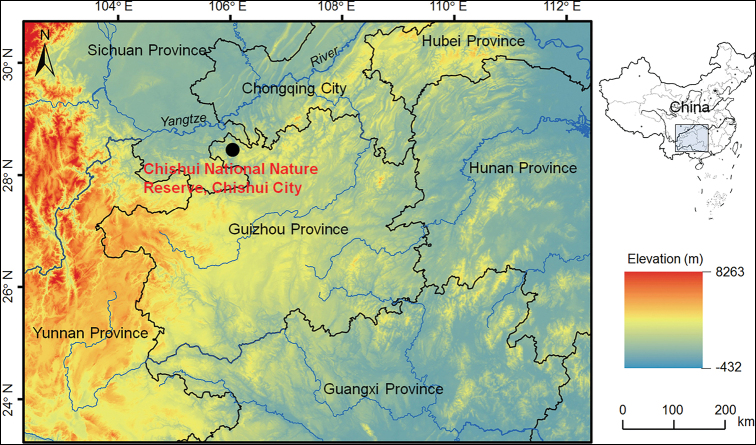
Location of the type locality of *Leptobrachella chishuiensis* sp. nov., Chishui National Nature Reserve, Chishui City, Guizhou Province, China.

**Specimens.** Seven adult males and one adult female of the undescribed species were collected from the mountain streams in Chishui National Nature Reserve, Chishui City, Guizhou Province, China (for voucher information see Table 1; Fig. 1). After taking photographs, they were euthanized using isoflurane, and then the specimens were fixed in 10% buffered formalin. Before fixing, muscle tissue was taken and preserved separately in 95% ethanol. Specimens were deposited in Chengdu Institute of Biology, Chinese Academy of Sciences (**CIB**, **CAS**).

**Molecular phylogenetic analyses.** All eight specimens of the new taxon were included in the molecular analyses (Table 1). For phylogenetic analyses, the corresponding gene sequences for all those related species for which comparable sequences were available were also downloaded from GenBank (Table 1) mainly based on previous studies (Chen et al. 2018; Wang et al. 2019; Luo et al. 2020). Corresponding sequences of *Leptobrachium tengchongense*, one *Leptobrachium huashen*, and one *Megophrys major* were also downloaded from GenBank, and used as outgroups according to previous phylogenetic works (Chen et al. 2018; Wang et al. 2019; Luo et al. 2020).

**Table 1.** Information for samples used in molecular phylogenetic analyses in this study.

ID	Species	Voucher	Locality	GenBank accession number
1	*Leptobrachella chishuiensis* sp. nov.	CIBCS20190518047	Chishui National Nature Reserve, Chishui City, Guizhou Province, China	MT117053
2	*Leptobrachella chishuiensis* sp. nov.	CIBCS20190518042	Chishui National Nature Reserve, Chishui City, Guizhou Province, China	MT117054
3	*Leptobrachella chishuiensis* sp. nov.	CIBCS20190518043	Chishui National Nature Reserve, Chishui City, Guizhou Province, China	MT117055
4	*Leptobrachella chishuiensis* sp. nov.	CIBCS20190518049	Chishui National Nature Reserve, Chishui City, Guizhou Province, China	MT117056
5	*Leptobrachella chishuiensis* sp. nov.	CIBCS20190518046	Chishui National Nature Reserve, Chishui City, Guizhou Province, China	MT117057
6	*Leptobrachella chishuiensis* sp. nov.	CIBCS20190518045	Chishui National Nature Reserve, Chishui City, Guizhou Province, China	MT117058
7	*Leptobrachella chishuiensis* sp. nov.	CIBCS20190518044	Chishui National Nature Reserve, Chishui City, Guizhou Province, China	MT330118
8	*Leptobrachella chishuiensis* sp. nov.	CIBCS20190518048	Chishui National Nature Reserve, Chishui City, Guizhou Province, China	MT330119
9	*Leptobrachella bijie*	SYS a007313/CIB110002	Mt. Zhaozi Nature Reserve, Bijie City, Guizhou Province, China	MK414532
10	*Leptobrachella bijie*	SYS a007314	Mt. Zhaozi Nature Reserve, Bijie City, Guizhou Province, China	MK414533
11	*Leptobrachella bijie*	SYS a007315	Mt. Zhaozi Nature Reserve, Bijie City, Guizhou Province, China	MK414534
12	*Leptobrachella purpuraventra*	SYS a007081	Wujing Nature Reserve, Bijie City, Guizhou Province, China	MK414517
13	*Leptobrachella purpuraventra*	SYS a007277/CIB110003	Wujing Nature Reserve, Bijie City, Guizhou Province, China	MK414518
14	*Leptobrachella purpuraventra*	SYS a007278	Wujing Nature Reserve, Bijie City, Guizhou Province, China	MK414519
15	*Leptobrachella suiyangensis*	GZNU20180606002	Huoqiuba Nature Reserve, Suiyang County, Guizhou, China	MK829648
16	*Leptobrachella suiyangensis*	GZNU20180606006	Huoqiuba Nature Reserve, Suiyang County, Guizhou, China	MK829649
17	*Leptobrachella suiyangensis*	GZNU20180606005	Huoqiuba Nature Reserve, Suiyang County, Guizhou, China	MK829650
18	*Leptobrachella purpura*	SYS a006530	Yingjiang County, Yunnan Province, China	MG520354
19	*Leptobrachella alpina*	KIZ046816	Huangcaoling, Yunnan Province, China	MH055866
20	*Leptobrachella bourreti*	AMS R 177673	Lao Cai Province, Vietnam	KR018124
21	*Leptobrachella oshanensis*	KIZ025776	Emei Shan, Emei Shan City, Sichuan Province, China	MH055895
22	*Leptobrachella eos*	MNHN:2004.0278	Phongsaly Province, Laos	JN848450
23	*Leptobrachella tengchongense*	SYS a004598	Tengchong County, Yunnan Province, China	KU589209
24	*Leptobrachella mangshanensis*	MSZTC201701	Mt. Mang, Yizhang County, Hunan Province, China	MG132196
25	*Leptobrachella liui*	SYS a001597	Mt. Wuyi, Wuyishan City, Fujian Provnce, China	KM014547
26	*Leptobrachella laui*	SYS a001507	Mt. Wutong, Shenzhen City, Guangdong Province, China	KM014544
27	*Leptobrachella yunkaiensis*	SYS a004664 / CIB107272	Dawuling Forest Station, Maoming City, Guangdong Province, China	MH605585
28	*Leptobrachella maoershanensis*	KIZ019385	Mt. Maoer Nature Reserve, Ziyuan County, Guangxi Province, China	KY986930
29	*Leptobrachella khasiorum*	SDBDU 2009.329	East Khasi Hills, Meghalaya, India	KY022303
30	*Leptobrachella yingjiangensis*	SYS a006532	Yingjiang County, Yunnan Province, China	MG520351
31	*Leptobrachella petrops*	AMS:R184826	Vietnam	KY459997
32	*Leptobrachella puhoatensis*	AMS:R184852	Pu Hoat Nature Reserve, Nghe An Province, Vietnam	KY849588
33	*Leptobrachella namdongensis*	VNUF A.2017.37	Thanh Hoa Provincen, Vietnam	MK965389
34	*Leptobrachella isos*	VNMN A 2015.4/AMS R 176480	Gia Lai Province, Vietnam	KT824769
35	*Leptobrachella firthi*	AMS R 176524	Kon Tum Province, Vietnam	JQ739206
36	*Leptobrachella minima*	KUHE:19201	Thailand	LC201981
37	*Leptobrachella ventripunctata*	SYS a004536	Zhushihe, Yunnan Province, China	MH055831
38	*Leptobrachella aerea*	ZFMK 86362	Quang Binh Provice, Vietnam	JN848409
39	*Leptobrachella wuhuangmontis*	SYS a003500 / CIB107274	Mt. Wuhuang, Pubei County, Guangxi Zhuang minority Autonomous Region, China	MH605581
40	*Leptobrachella pluvialis*	MNHN:1999.5675	Mt. Fan Si Pan, Lao Cai Province, Vietnam	JN848391
41	*Leptobrachella shangsiensis*	NHMG1704003	Shangsi County, Guangxi Zhuang minority Autonomous Region, China	MK095463
42	*Leptobrachella nahangensis*	ROM 7035	Na Hang Nature Reserve, Tuyen Quang, Vietnam	MH055853
43	*Leptobrachella nyx*	AMNH A163810	Ha Giang Province, Vietnam	DQ283381
44	*Leptobrachella zhangyapingi*	KIZ07258	Pang Num Poo, Chiang Mai Province, Thailand	MH055864
45	*Leptobrachella sungi*	ROM 20236	Tam Dao, Vinh Phuc, Vietnam	MH055858
46	*Leptobrachella tuberosa*	ZMMU-NAP-02275	Kon Ka Kinh National Park, Gia Lai, Vietnam	MH055959
47	*Leptobrachella botsfordi*	VNMN 03682	Fansipan, Lao Cai, Vietnam	MH055953
48	*Leptobrachella pallida*	UNS00510	Lam Dong Province, Vietnam	KR018112
49	*Leptobrachella kalonensis*	IEBR A.2015.15	Binh Thuan Province, Vietnam	KR018114
50	*Leptobrachella bidoupensis*	NAP-01453	Lam Dong Province, Vietnam	KP017573
51	*Leptobrachella tadungensis*	UNS00515	Dak Nong Province, Vietnam	KR018121
52	*Leptobrachella maculosa*	AMS R 177660	Ninh Thuan Province, Vietnam	KR018119
53	*Leptobrachella pyrrhops*	ZMMU ABV-00148	Loc Bao, Lam Dong Provice, Vietnam	KP017575
54	*Leptobrachella macrops*	IEBR A.2017.9	Hon Den Mt., Phu Yen Province, Vietnam	MG787990
55	*Leptobrachella melica*	MVZ 258197	Virachey National Park, Ratanakiri Province, Cambodia	HM133599
56	*Leptobrachella applebyi*	AMS R171704	Song Thanh, Quang Nam, Vietnam	HM133598
57	*Leptobrachella rowleyae*	ITBCZ 2783	Son Tra, Da Nang City, Vietnam	MG682552
58	*Leptobrachella ardens*	AMS R 176463	Gia Lai Province, Vietnam	KR018110
59	*Leptobrachella crocea*	AMS R 173740	Kon Tum, Vietnam	MH055954
60	*Leptobrachella melanoleuca*	KUHE 23840	Srat Thani, Thailand	LC201997
61	*Leptobrachella fuliginosa*	KUHE:20172	Thailand	LC201985
62	*Leptobrachella itiokai*	KUHE:55897	Mulu NP, Sarawak, Borneo, Malaysia	LC137805
63	*Leptobrachella brevicrus*	ZMH A09365	Sarawak: Gunung Mulu National Park: Small stream of the Sungei Tapin, Malaysia	KJ831302
64	*Leptobrachella parva*	KUHE 55308	Mulu NP, Sarawak, Borneo, Malaysia	LC056791
65	*Leptobrachella baluensis*	SP 21604	Tambunan, Sabah, Borneo, Malaysia	LC056792
66	*Leptobrachella mjobergi*	KUHE 17064	Gading NP, Sarawak, Borneo, Malaysia	LC056785
67	*Leptobrachella juliandringi*	SRC 00230/KUHE 49815	Mulu NP, Sarawak, Borneo, Malaysia	LC056779
68	*Leptobrachella arayai*	BORNEEISIS 22931	Liwagu, Kinabalu, Borneo, Malaysia	AB847558
69	*Leptobrachella hamidi*	KUHE 17545	Borneo, Malaysia	AB969286
70	*Leptobrachella marmorata*	KUHE 53227	Annah Rais, Padawan, Kuching Division, Sarawak, Malaysia	AB969289
71	*Leptobrachella maura*	SP 21450	Kinabalu, Sabah, Malaysia	AB847559
72	*Leptobrachella gracilis*	KUHE 55624	Camp 1, Gunung Mulu, Borneo, Malaysia	AB847560
73	*Leptobrachella sabahmontana*	BORNEENSIS 12632	Borneo, Malaysia	AB847551
74	*Leptobrachella dringi*	KUHE 55610	Camp 4 of Gunung Mulu, Malaysia	AB847553
75	*Leptobrachella picta*	UNIMAS 8705	Borneo, Malaysia	KJ831295
76	*Leptobrachella fritinniens*	KUHE 55371	Headquarters, Gunung Mulu, Malaysia	AB847557
77	*Leptobrachella sola*	KUHE 23261	Hala Bala, Thailand	LC202007
78	*Leptobrachella heteropus*	KUHE 15487	Larut, Peninsular, Malaysia	AB530453
79	*Leptobrachella kecil*	KUHE 52440	Malaysia	LC202004
80	*Leptobrachella kajangensis*	LSUHC 4439	Tioman, Malaysia	LC202002
81	*Leptobrachium tengchongense*	SYSa004604d	Yunnan Province, China	KX066880
82	*Leptobrachium huashen*	KIZ049025	Yunnan Province, China	KX811931
83	*Megophrys major*	AMS R 173870	Kon Tum, Vietnam	KY476333

Total DNA was extracted using a standard phenol-chloroform extraction protocol (Sambrook et al. 1989). The mitochondrial 16S rRNA gene (16S) sequences were amplified, and the primers P7 (5’-CGCCTGTTTACCAAAAACAT-3’) and P8 (5’-CCGGTCTGAACTCAGATCACGT-3’) were used following Simon et al. (1994). Gene fragments were amplified under the following conditions: an initial denaturing step at 95 °C for 4 min; 36 cycles of denaturing at 95 °C for 30 s, annealing at 51 °C for 30 s and extending at 72 °C for 70 s. Sequencing was conducted using an ABI3730 automated DNA sequencer in Shanghai DNA BioTechnologies Co., Ltd. (Shanghai, China). New sequences were deposited in GenBank (for GenBank accession numbers see Table 1).

Sequences were assembled and aligned using the Clustalw module in BioEdit v. 7.0.9.0 (Hall 1999) with default settings. Phylogenetic analyses were conducted using Maximum Likelihood (ML) and Bayesian Inference (BI) methods, implemented in PhyML v. 3.0 (Guindon et al. 2010) and MrBayes v. 3.12 (Ronquist and Huelsenbeck 2003), respectively. We ran JMODELTEST v. 2.1.2 (Darriba et al. 2012) with Akaike and Bayesian information criteria on the alignment, resulting in the best-fitting nucleotide substitution models of GTR + I + G for the data used in ML and BI analyses. For the ML analysis, branch supports were drawn from 10,000 nonparametric bootstrap replicates. In BI analysis, the parameters for each partition were unlinked, and branch lengths were allowed to vary proportionately across partitions. Two runs each with four Markov chains were simultaneously run for 60 million generations with sampling every 1,000 generations. The first 25% trees were removed as the “burn-in” stage followed by calculations of Bayesian posterior probabilities and the 50% majority-rule consensus of the post burn-in trees sampled at stationarity. Finally, genetic distance between *Leptobrachella* species based on uncorrected *p*-distance model was estimated on 16S gene using MEGA v. 6.06 (Tamura et al. 2013).

**Morphological comparisons.** All eight adult specimens (Table 2) of the new taxon were measured. The terminology and methods followed Fei et al. (2005), Mahony et al. (2011), and Wang et al. (2019). Measurements were made with a dial caliper to the nearest 0.1 mm (Watters et al. 2016) with digital calipers. Corresponding measurements of *L. bijie* and *L. purpuraventra* were retrieved from Wang et al. (2019). Twenty-three morphometric characters of adult specimens were measured:

**ED** eye diameter (distance from the anterior corner to the posterior corner of the eye);

**FIL** first finger length (distance from base to tip of finger I);

**FIIL** second finger length (distance from base to tip of finger II);

**FIIIL** third finger length (distance from base to tip of finger III);

**FIVL** fourth finger length (distance from base to tip of finger IV);

**FL** foot length (distance from tarsus to the tip of the fourth toe);

**HDL** head length (distance from the tip of the snout to the articulation of jaw);

**HDW** head width (greatest width between the left and right articulations of jaw);

**HLL** hindlimb length (distance from tip of fourth toe to vent);

**IND** internasal distance (minimum distance between the inner margins of the external nares);

**IOD** interorbital distance (minimum distance between the inner edges of the upper eyelids);

**LAL** length of lower arm and hand (distance from the elbow to the distal end of the Finger IV);

**LW** lower arm width (maximum width of the lower arm);

**ML** manus length (distance from tip of third digit to proximal edge of inner palmar tubercle);

**SL** snout length (distance from the tip of the snout to the anterior corner of the eye);

**SVL** snout-vent length (distance from the tip of the snout to the posterior edge of the vent);

**TYD** maximal tympanum diameter;

**TEY** tympanum-eye distance (distance from anterior edge of tympanum to posterior corner of eye);

**TFL** length of foot and tarsus (distance from the tibiotarsal articulation to the distal end of the toe IV);

**THL** thigh length (distance from vent to knee);

**TL** tibia length (distance from knee to tarsus);

**TW** maximal tibia width;

**UEW** upper eyelid width (greatest width of the upper eyelid margins measured perpendicular to the anterior-posterior axis).

In order to reduce the impact of allometry, the correct value from the ratio of each character to SVL was calculated and then was log-transformed for the following morphometric analyses. Mann-Whitney *U* tests were conducted to test the significance of differences on morphometric characters between the undescribed species, *L. bijie* and *L. purpuraventra*. The significance level was set at 0.05. Furthermore, principal component analyses (PCA) were conducted to highlight whether the different species were separated in morphometric space. Due to only the measurements SVL, HDL, HDW, SL, IND, IOD, ED, TYD, TEY, LAL, ML, TL, HLL, and FL of male *L. bijie* and *L. purpuraventra* being available from Wang et al. (2019), the morphometric analyses were conducted only based on these 14 morphometric characters for male group.

The new taxon was also compared with all other congeners of *Leptobrachella* based on morphological characters. Comparative morphological data were obtained from literatures (Table 3).

**Bioacoustics analyses.** The advertisement calls of the new taxon were recorded from the holotype specimen CIBCS20190518047 in the field on 18 May 2019 in Chishui National Nature Reserve, Chishui City, Guizhou Province, China. The advertisement call of the new species was recorded in the stream at ambient air temperature of 20 °C and air humidity of 87%. SONY PCM-D50 digital sound recorder was used to record within 20 cm of the calling individual. The sound files in wave format were resampled at 48 kHz with sampling depth 24 bits. Calls were recoded and examined as described by Wijayathilaka and Meegaskumbura (2016). Call recordings were visualized and edited with SoundRuler v. 0.9.6.0 (Gridi-Papp 2003–2007) and Raven Pro v. 1.5 software (Cornell Laboratory of Ornithology, Ithaca, NY, USA). Ambient temperature of the type locality was taken by a digital hygrothermograph.

## Results

**Figure 2.**
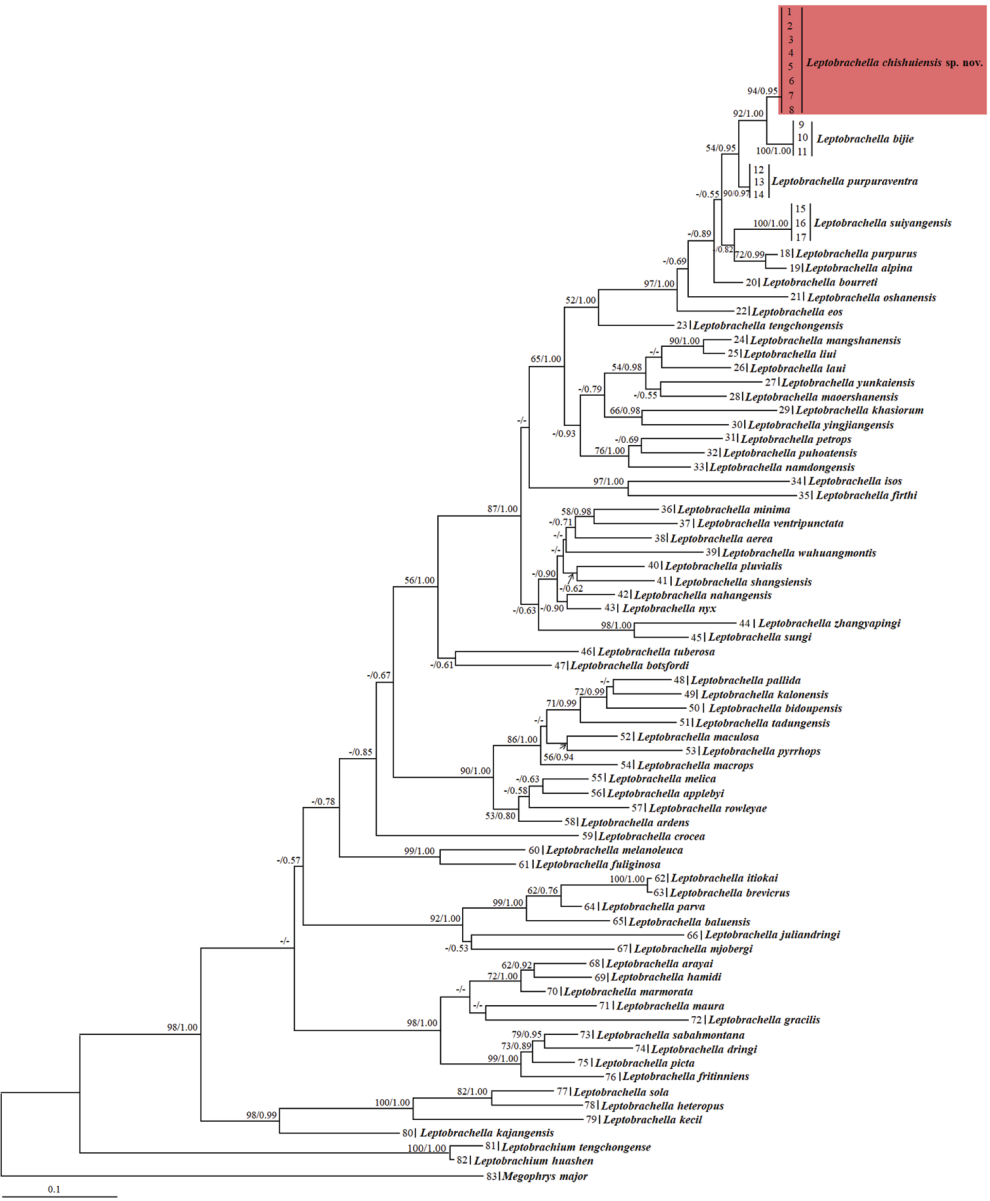
Maximum Likelihood (ML) tree based on the mitochondrial 16S rRNA sequences. Bootstrap supports from ML analyses/Bayesian posterior probabilities from Bayesian Inference (BI) analyses are labelled beside nodes. Information of samples 1–83 refer to Table 1.

Aligned sequence matrix of 16S contained 537 bps. ML and BI analyses based on the 16S matrix resulted in essentially identical topologies (Fig. 2). All six samples of the new taxon were clustered into one monophyletic group (node supports in ML and BI: 94 and 0.95) nested into *Leptobrachella*, and was a sister taxon to *L. bijie* (node supports in ML and BI: 92 and 1.00). The genetic distance between the new taxon and its closest relatives *L. bijie* was 2.1%, at the same level with that between *L. alpina* and *L. purpura* (2.1%; Suppl. material 1: Table S1).

**Figure 3.**
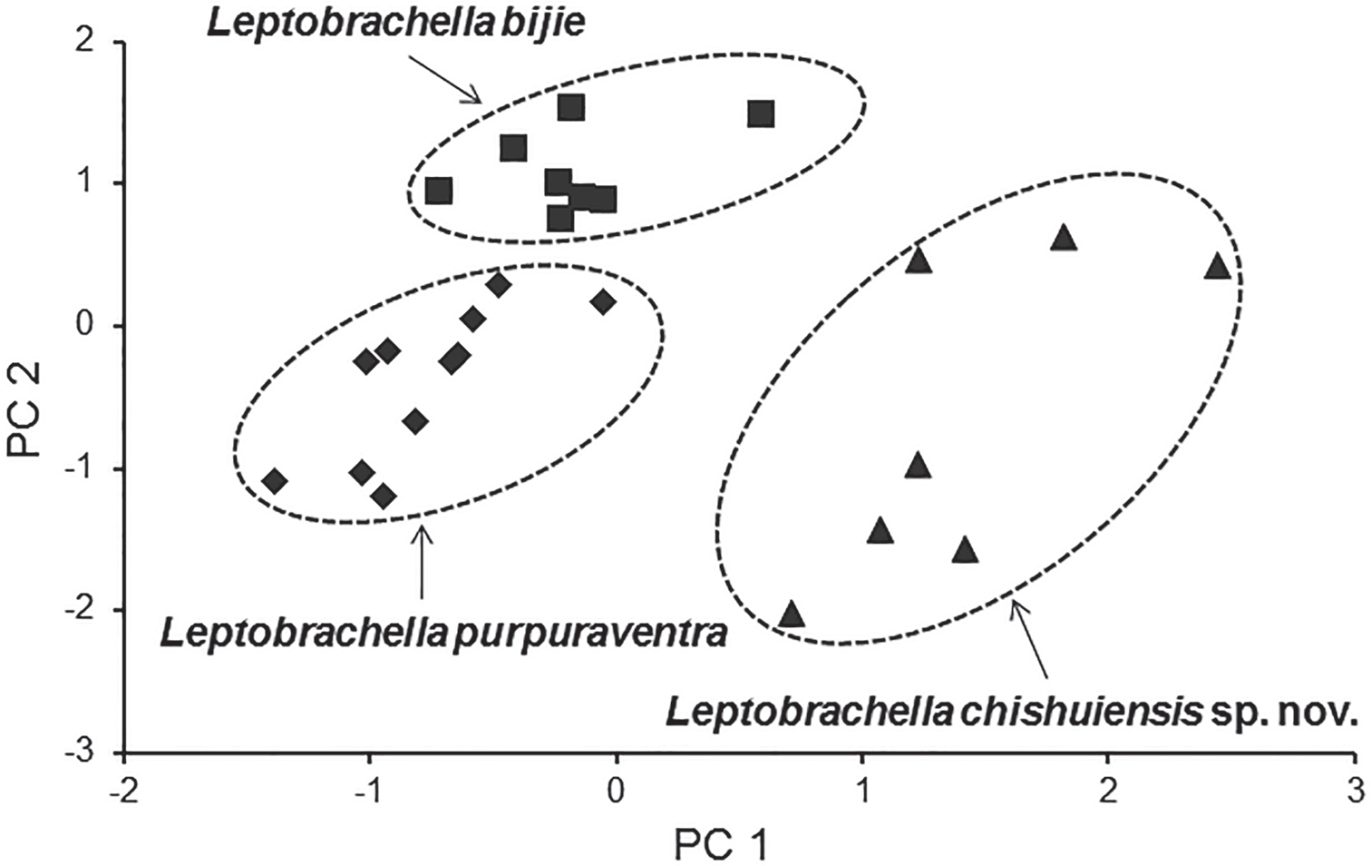
Plots of the first principal component (PC1) versus the second (PC2) for *Leptobrachella chishuiensis* sp. nov., *L. bijie*, and *L. purpuraventra* in males from a principal component analysis.

In PCA for male group, the total variation of the first two principal components was 64.6%. In males on the two-dimensional plots of PC1 vs. PC2, the undescribed species could be distinctly separated from *L. bijie* and *L. purpuraventra* (Fig. 3). The results of Mann-Whitney *U* tests indicated that in males, the new taxon was significantly different from *L. bijie* and *L. purpuraventra* on many morphometric characters (all *p*-values < 0.05; Table 4).

There were many differences in sonograms and waveforms of calls between the new species *L. bijie*, and *L. purpuraventra*. Firstly, a call contains 1–4 notes in the new species and only contains two notes of each call in *L. bijie* and *L. purpuraventra*. Secondly, the dominant frequency of the new species is higher than *L. bijie* and *L. purpuraventra*.

Based on the molecular, morphological, and bioacoustics differences, the specimens from Chishui City, Guizhou Province, China represent a new species which is described as follows.

## Taxonomic account

### Leptobrachella chishuiensis sp. nov.

Taxon classificationAnimaliaAnuraMegophryidae

B36388D0-D16F-5087-8F4F-A957553F423C

http://zoobank.org/DE8BA5C5-CB7B-4872-B489-61E7EFCF9B8C

**Figure 4.**
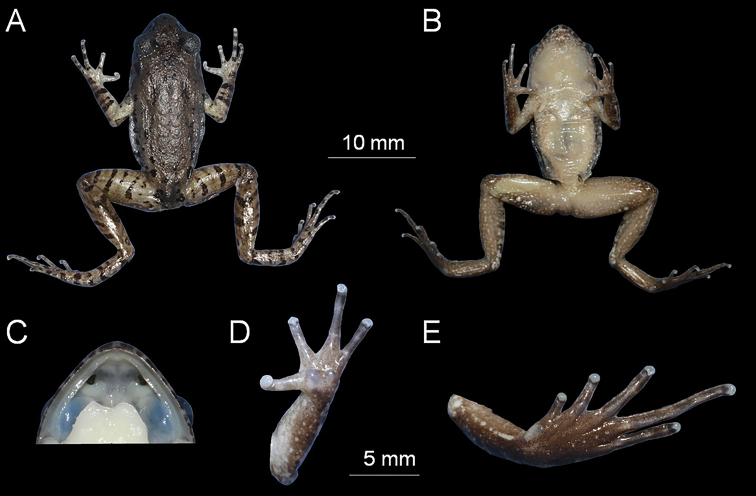
The holotype specimen CIBCS20190518047 of *Leptobrachella chishuiensis* sp. nov. in preservative **A** dorsal view **B** ventral view **C** frontal view of tongue **D** ventral view of hand **E** ventral view of foot.

**Figure 5.**
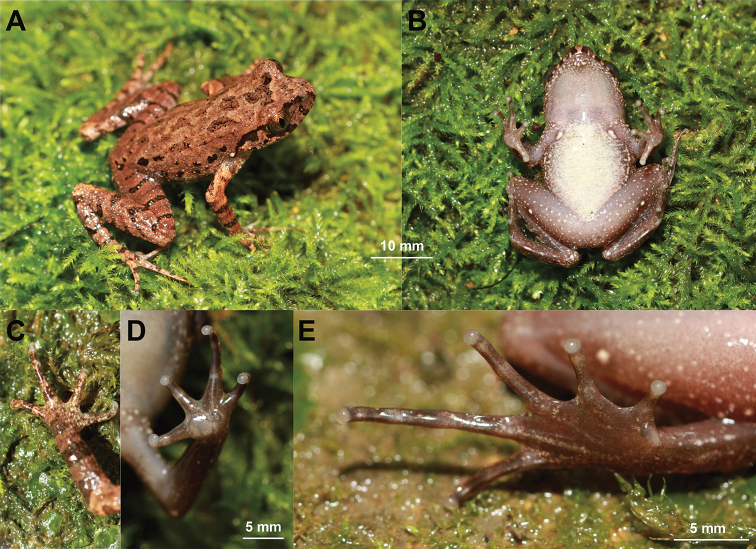
Photos of the holotype CIBCS20190518047 of *Leptobrachella chishuiensis* sp. nov. in life **A** dorsal view **B** ventral view **C** dorsal view of hand **D** ventral view of hand **E** ventral view of foot.

**Figure 6.**
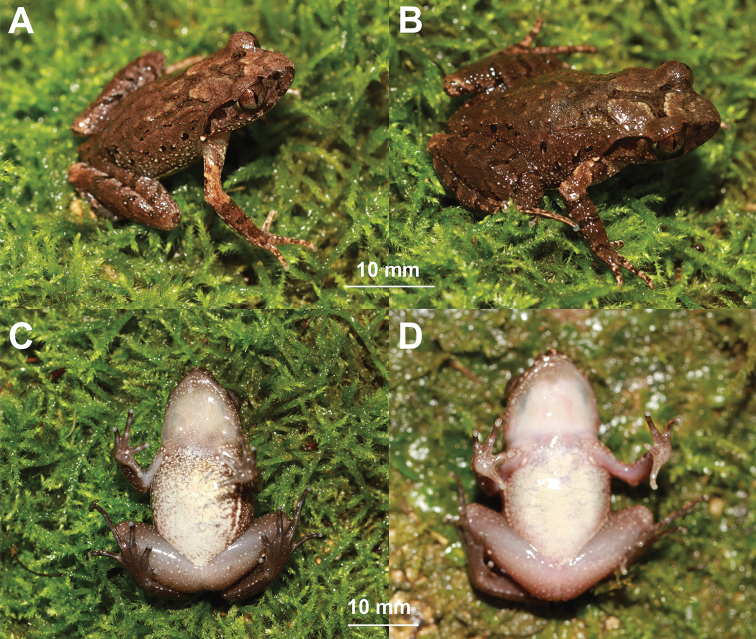
Colour variation in *Leptobrachella chishuiensis* sp. nov. **A** dorsal view of the male specimen CIBCS20190518042 **B** dorsal view of the female specimen CIBCS20190518046 **C** ventral view of the female specimen CIBCS20190518046 **D** ventral view of the female specimen CIBCS20190518049.

Figs 4 , 5 , 6 ; Tables 1 , 2 , 3 , 4 , 5

#### Type material.

***Holotype*.** CIBCS20190518047, adult male (Figs 4, 5), collected by Shi-Ze Li in Chishui National Nature Reserve (28.436708N, 105.997794E, ca. 465 m a. s. l.), Chishui City, Guizhou Province, China on 18 May 2019.

***Paratypes*.** Six adult males and one adult female from Chishui City, Guizhou Province, China, collected by Shize LI and Jing LIU. One female CIBCS20190518046 and two adult males CIBCS 20190518048 and CIBCS20190518049 collected by Jing LIU on 18 May 2019, four adult males CIBCS 20190518042, CIBCS 20190518043, CIBCS20190518044 and CIBCS20190518045 collected by Shize LI on 18 May 2019.

#### Diagnosis.

*Leptobrachella chishuiensis* sp. nov. is assigned to the genus *Leptobrachella* based on molecular phylogenetic analyses and the following morphological characters: small body size; having an elevated inner metacarpal tubercle; having macro-glands on body (including supra-axillary, femoral andventrolateral glands); lacking vomerine teeth; having small tubercles on eyelids; anterior tip of snout with whitish vertical bar (Dubois 1983; Matsui 1997, 2006; Lathrop et al. 1998; Delorme et al. 2006; Das et al. 2010; Luo et al. 2020).

*Leptobrachella chishuiensis* sp. nov. could be distinguished from its congeners by a combination of the following characters: (1) small body size (SVL 30.8–33.4 mm in seven adult males, and 34.2 mm in one adult female); (2) dorsal skin shagreened, some of the granules forming longitudinal short skin ridges; (3) tympanum distinctly discernible, slightly concave; (4) internasal distance longer than interorbital distance; (5) supra-axillary, femoral, pectoral and ventrolateral glands distinctly visible; (6) absence of webbing and lateral fringes on fingers; (7) toes with rudimentary webbing and shallow lateral fringes; (8) relative finger lengths II < IV < I < III; (9) heels overlapped when thighs are positioned at right angles to the body; and (10) tibia-tarsal articulation reaches the tympanum.

#### Description of holotype.

Measurements in mm. Adult male (CIBCS20190518047). SVL 32.4. Head length slightly longer than head width (HDL/HDW ratio 1.04); snout slightly protruding, projecting slightly beyond margin of the lower jaw; nostril closer to snout than eye; canthus rostralis gently rounded; loreal region slightly concave; interorbital space ﬂat, internarial distance longer than interorbital distance (IND/IOD ratio 1.23); pineal ocellus absent; vertical pupil; snout length larger than eye diameter; tympanum distinct, rounded, and slightly concave, diameter smaller than that of the eye (TMP/ED ratio 0.57); upper margin of tympanum in contact with supratympanic ridge; distinct black supratympanic line present; vomerine teeth absent; tongue notched behind; supratympanic ridge distinct, extending from posterior corner of eye to supra-axillary gland.

Tips of fingers rounded, slightly swollen; relative finger lengths II < IV < I < III (FIL/FIIL ratio 1.1, FIVL/FIIL ratio 1.03); absence of webbing; nuptial pad and subarticular tubercles absent; inner palmar tubercle large, rounded separated from small, round outer palmar tubercle.

Hindlimbs slender, tibia 49% of snout-vent length; heels overlapped when thighs are positioned at right angles to the body, tibiotarsal articulation reaching tympanum when leg stretched forward; tibia length slightly longer than thigh length; relative toe lengths I < II < V < III < IV; tips of toes rounded, slightly dilated; subarticular tubercle small, distinct at the base of each toes; toes without webbing; narrow lateral fringes present on all toes; inner metatarsal tubercle present, large, oval, outer metatarsal tubercle absent; dorsal surface shagreened and granular, some of the granules forming short longitudinal folds on the flank of dorsal; ventral skin smooth; dense tiny granules present on surface of chest and ventral surface of thigh and tibia; pectoral gland and femoral gland oval, distinctly visible. Ventrolateral gland distinctly visible and forming an incomplete line.

#### Colouration of holotype in life.

Dorsum brown, with small, distinct darker brown markings and spots and scattered with irregular light orange pigmentation. A dark brown inverted triangular pattern between anterior corner of eyes. Tympanum brown, a dark brown bar above tympanum, and a dark brown bar under the eye; transverse dark brown bars on dorsal surface of limbs; distinct dark brown blotches on ﬂanks from groin to axilla, longitudinally in two rows; elbow and upper arms with dark bars and distinct coppery orange coloration; fingers and toes with distinct dark bars. Ventral surface of throat grey purple, chest and belly white, presence of distinct nebulous greyish speckling on ﬂanks; ventral surface of limbs grey purple. Supra-axillary gland, femoral, pectoral and ventrolateral glands white (Fig. 5).

#### Preserved holotype colouration.

Dorsum of body and limbs fade to dark brown; transverse bars on limbs become more distinct ventral surface of body and limbs fade to greyish white. Supra-axillary, femoral, pectoral and ventrolateral glands fade to greyish white (Fig. 4).

#### Variations.

Morphological measurements were showed in Table 2. All specimens were similar in morphology but some individuals different from the holotype in color pattern. In some adult males, a dark brown inverted triangular pattern between anterior corner of eyes, in connected to the dark brown W-shaped marking on interorbital region (Fig. 6A); in adult female, the color of dorsum is blacker (Fig. 6B) and some patchiness on the chest and the flank of belly (Fig. 6C); in some adult males, the throat and bell creamy and white patchiness sparse on the ventral surface of limbs (Fig. 6D); in some specimens, the tibiotarsal articulation reaching tympanum to eye when leg stretched forward.

**Table 2.** Measurements of *Leptobrachella chishuiensis* sp. nov. Units in mm. See abbreviations for characters in the Materials and methods section.

Species	Voucher number	Sex	SVL	HDL	HDW	SL	IND	IOD	UEW	ED	TYD	LAL	LW	THL	TW	TL	TFL	FL	FIL	FIIL	FIIIL	FIVL
*Leptobrachella chishuiensis* sp. nov.	CIBCS20190518047	male	32.4	12.3	11.8	5.1	3.8	3.1	3.3	4.6	2.6	17.0	3.2	16.0	4.3	16.2	22.3	15.6	3.4	3.1	5.0	3.2
*Leptobrachella chishuiensis* sp. nov.	CIBCS20190518042	male	32.7	12.2	11.9	5.8	3.5	3.1	3.1	5.0	2.2	15.4	3.1	15.3	3.6	15.5	22.3	14.7	3.6	3.4	5.5	3.5
*Leptobrachella chishuiensis* sp. nov.	CIBCS20190518043	male	33.0	11.9	11.7	5.1	3.5	2.8	3.0	4.0	2.2	15.3	3.1	15.2	4.2	15.5	22.2	15.3	3.3	3.0	4.9	3.2
*Leptobrachella chishuiensis* sp. nov.	CIBCS20190518049	male	30.9	11.9	10.8	5.0	3.5	3.0	3.1	4.1	2.2	14.9	2.6	13.9	3.4	15.3	21.1	14.4	3.0	2.8	5.1	2.9
*Leptobrachella chishuiensis* sp. nov.	CIBCS20190518044	male	33.4	11.1	11.6	5.4	3.8	3.1	3.3	4.4	2.2	16.3	2.8	17.1	3.8	16.8	22.1	15.9	3.8	3.0	5.0	3.5
*Leptobrachella chishuiensis* sp. nov.	CIBCS20190518045	male	30.8	11.8	11.4	4.8	3.6	3.0	3.0	4.1	2.0	15.5	3.0	14.2	4.1	15.2	21.2	15.1	3.6	3.1	5.0	3.1
*Leptobrachella chishuiensis* sp. nov.	CIBCS20190518048	male	31.6	11.5	10.6	5.0	3.7	2.7	3.3	4.2	2.6	14.7	2.9	13.7	3.3	14.9	20.9	15.0	3.2	2.8	5.0	2.9
*Leptobrachella chishuiensis* sp. nov.	CIBCS20190518046	female	34.2	12.7	12.0	5.3	3.4	2.7	3.0	4.4	2.4	16.3	3.3	15.3	4.2	16.0	22.2	16.3	3.4	3.0	5.6	3.3

#### Advertisement call.

**Figure 7.**
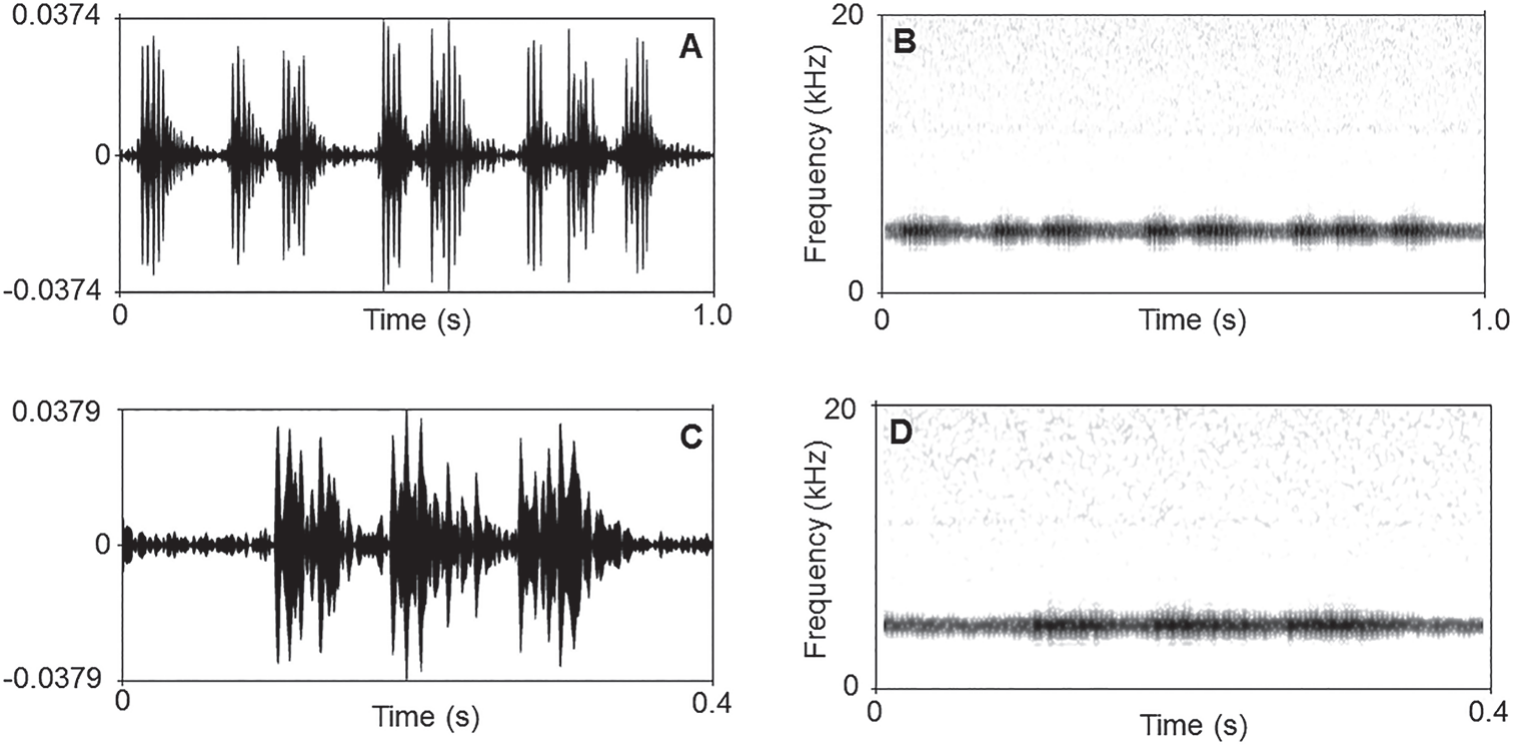
Advertisement calls of the holotype CIBCS20190518047 of *Leptobrachella chishuiensis* sp. nov. **A** waveform showing one second contains 4 calls **B** sonogram showing one second contains 4 calls **C** waveform showing 0.4 second contains a call **D** sonogram showing 0.4 second contains a call.

A total of 32 advertisement calls of *Leptobrachella chishuiensis* sp. nov. were recorded in Chishui City, Guizhou Province, China on 18 May 2019 between 21:00–22:00. The call description is based on recordings of the holotype CIBCS20190518047 (Fig. 7) from a branch of bush nearby a stream. Each call contains 1–4 notes (mean 2.34 ± 0.827, *N* = 32). Call duration was 75–353 ms (mean 200 ± 67, *N* = 32). Call interval was 8–98 ms (mean 60 ± 21, *N* = 31) with a peak frequency was 6140.15 ± 69.35 (6064–6284 Hz, *N* = 32). Each note had a duration of 52–950 ms (mean 104 ± 107, *N* = 69), and the intervals between notes had a duration of 0. 1–25 ms (mean 5.3 ± 8.5, *N* = 37). Amplitude modulation within note was apparent, beginning with high energy pulses then decreasing towards the end of each note.

**Table 3.** References for morphological characters for congeners of the genus *Leptobrachella*.

ID	*Leptobrachella* species	Literature obtained
1	*L. aerea* (Rowley, Stuart, Richards, Phimmachak & Sivongxay, 2010)	Rowley et al. 2010c
2	*L. alpina* (Fei, Ye & Li, 1990)	Fei et al. 2009
3	*L. applebyi* (Rowley & Cao, 2009)	Rowley and Cao 2009
4	*L. arayai* (Matsui, 1997)	Matsui 1997
5	*L. ardens* (Rowley, Tran, Le, Dau, Peloso, Nguyen, Hoang, Nguyen & Ziegler, 2016)	Rowley et al. 2016
6	*L. baluensis* Smith, 1931	Dring 1983; Eto et al. 2016
7	*L. bidoupensis* (Rowley, Le, Tran & Hoang, 2011)	Rowley et al. 2011
8	*L. bijie* Wang, Li, Li, Chen & Wang, 2019	Wang et al. 2019
9	*L. bondangensis* Eto, Matsui, Hamidy, Munir & Iskandar, 2018	Eto et al. 2018
10	*L. botsfordi* (Rowley, Dau & Nguyen, 2013)	Rowley et al. 2013
11	*L. bourreti* (Dubois, 1983)	Ohler et al. 2011
12	*L. brevicrus* Dring, 1983	Dring 1983; Eto et al. 2015
13	*L. crocea* (Rowley, Hoang, Le, Dau & Cao, 2010)	Rowley et al. 2010a
14	*L. dringi* (Dubois, 1987)	Inger et al. 1995; Matsui and Dehling 2012
15	*L. eos* (Ohler, Wollenberg, Grosjean, Hendrix, Vences, Ziegler & Dubois, 2011)	Ohler et al. 2011
16	*L. firthi* (Rowley, Hoang, Dau, Le & Cao, 2012)	Rowley et al. 2012
17	*L. fritinniens* (Dehling & Matsui, 2013)	Dehling and Matsui 2013
18	*L. fuliginosa* (Matsui, 2006)	Matsui 2006
19	*L. fusca* Eto, Matsui, Hamidy, Munir & Iskandar, 2018	Eto et al. 2018
20	*L. gracilis* (Günther, 1872)	Günther 1872; Dehling 2012b
21	*L. hamidi* (Matsui, 1997)	Matsui 1997
22	*L. heteropus* (Boulenger, 1900)	Boulenger 1900
23	*L. isos* (Rowley, Stuart, Neang, Hoang, Dau, Nguyen & Emmett, 2015)	Rowley et al. 2015a
24	*L. itiokai* Eto, Matsui & Nishikawa, 2016	Eto et al. 2016
25	*L. juliandringi* Eto, Matsui & Nishikawa, 2015	Eto et al. 2015
26	*L. kajangensis* (Grismer, Grismer & Youmans, 2004)	Grismer et al. 2004
27	*L. kalonensis* (Rowley, Tran, Le, Dau, Peloso, Nguyen, Hoang, Nguyen & Ziegler, 2016)	Rowley et al. 2016
28	*L. kecil* (Matsui, Belabut, Ahmad & Yong, 2009)	Matsui et al. 2009
29	*L. khasiorum* (Das, Tron, Rangad & Hooroo, 2010)	Das et al. 2010
30	*L. lateralis* (Anderson, 1871)	Anderson 1871; Humtsoe et al. 2008
31	*L. laui* (Sung, Yang & Wang, 2014)	Sung et al. 2014
32	*L. liui* (Fei & Ye, 1990)	Fei et al. 2009; Sung et al. 2014
33	*L. macrops* (Duong, Do, Ngo, Nguyen & Poyarkov, 2018)	Duong et al. 2018
34	*L. maculosa* (Rowley, Tran, Le, Dau, Peloso, Nguyen, Hoang, Nguyen & Ziegler, 2016)	Rowley et al. 2016
35	*L. mangshanensis* (Hou, Zhang, Hu, Li, Shi, Chen, Mo, & Wang, 2018)	Hou et al. 2018
36	*L. maoershanensis* (Yuan, Sun, Chen, Rowley & Che, 2017)	Yuan et al. 2017
37	*L. marmorata* (Matsui, Zainudin & Nishikawa, 2014)	Matsui et al. 2014b
38	*L. maura* (Inger, Lakim, Biun & Yambun, 1997)	Inger et al. 1997
39	*L. melanoleuca* (Matsui, 2006)	Matsui 2006
40	*L. melica* (Rowley, Stuart, Neang & Emmett, 2010)	Rowley et al. 2010b
41	*L. minima* (Taylor, 1962)	Taylor 1962; Ohler et al. 2011
42	*L. mjobergi* (Smith, 1925)	Eto et al. 2015
43	*L. namdongensis* (Hoang, Nguyen, Luu, Nguyen & Jiang, 2019)	Hoang et al. 2019
44	*L. nahangensis* (Lathrop, Murphy, Orlov & Ho, 1998)	Lathrop et al. 1998
45	*L. natunae* (Günther, 1895)	Günther 1895
46	*L. nokrekensis* (Mathew & Sen, 2010)	Mathew and Sen 2010
47	*L. nyx* (Ohler, Wollenberg, Grosjean, Hendrix, Vences, Ziegler & Dubois, 2011)	Ohler et al. 2011
48	*L. oshanensis* (Liu, 1950)	Fei et al. 2009
49	*L. pallida* (Rowley, Tran, Le, Dau, Peloso, Nguyen, Hoang, Nguyen & Ziegler, 2016)	Rowley et al. 2016
50	*L. palmata* Inger & Stuebing, 1992	Inger and Stuebing 1992
51	*L. parva* Dring, 1983	Dring 1983
52	*L. pelodytoides* (Boulenger, 1893)	Boulenger 1893; Ohler et al. 2011
53	*L. petrops* (Rowley, Dau, Hoang, Le, Cutajar & Nguyen, 2017)	Rowley et al. 2017a
54	*L. picta* (Malkmus, 1992)	Malkmus 1992
55	*L. platycephala* (Dehling, 2012)	Dehling 2012a
56	*L. pluvialis* (Ohler, Marquis, Swan & Grosjean, 2000)	Ohler et al. 2000, 2011
57	*L. puhoatensis* (Rowley, Dau & Cao, 2017)	Rowley et al. 2017b
58	*L. purpuraventra* Wang, Li, Li, Chen & Wang, 2019	Wang et al. 2019
59	*L. purpura* (Yang, Zeng & Wang, 2018)	Yang et al. 2018
60	*L. pyrrhops* (Poyarkov, Rowley, Gogoleva, Vassilieva, Galoyan & Orlov, 2015)	Poyarkov et al. 2015
61	*L. rowleyae* (Nguyen, Poyarkov, Le, Vo, Ninh, Duong, Murphy & Sang, 2018)	Nguyen et al. 2018
62	*L. sabahmontana* (Matsui, Nishikawa & Yambun, 2014)	Matsui et al. 2014a
63	*L. serasanae* Dring, 1983	Dring 1983
64	*L. shangsiensis* Chen, Liao, Zhou & Mo, 2019	Chen et al. 2019
65	*L. sola* (Matsui, 2006)	Matsui 2006
66	*L. sungi* (Lathrop, Murphy, Orlov & Ho, 1998)	Lathrop et al. 1998
67	*L. suiyangensis* (Luo, Xiao, Gao & Zhou, 2020)	Luo et al. 2020
68	*L. tadungensis* (Rowley, Tran, Le, Dau, Peloso, Nguyen, Hoang, Nguyen & Ziegler, 2016)	Rowley et al. 2016
69	*L. tamdil* (Sengupta, Sailo, Lalremsanga, Das & Das, 2010)	Sengupta et al. 2010
70	*L. tengchongense* (Yang, Wang, Chen & Rao, 2016)	Yang et al. 2016
71	*L. tuberosa* (Inger, Orlov & Darevsky, 1999)	Inger et al. 1999
72	*L. ventripunctata* (Fei, Ye & Li, 1990)	Fei et al. 2009
73	*L. wuhuangmontis* Wang, Yang & Wang, 2018	Wang et al. 2018
74	*L. yingjiangensis* (Yang, Zeng & Wang, 2018)	Yang et al. 2018
75	*L. yunkaiensis* Wang, Li, Lyu & Wang, 2018	Wang et al. 2018
76	*L. zhangyapingi* (Jiang, Yan, Suwannapoom, Chomdej & Che, 2013)	Jiang et al. 2013

#### Secondary sexual characteristics.

Adult males with a large subgular vocal sac, and nupital pads and spines absent.

#### Comparisons.

The new species was compared with 52 congeners on morphology (Table 4). By having small body size (SVL 30.8–33.4 mm in seven adult males, and 34.2 mm in one adult female), *Leptobrachella chishuiensis* sp. nov. differs from the larger *L. bourreti* (42.0–45.0 mm in females), *L. eos* (33.1–34.7 mm in males and 40.7 in female), *L. lateralis* (36.6 mm in females), *L. nahangensis* (40.8 mm in male), *L. nyx* (37.0–41.0 mm in females), *L. platycephala* (35.1 mm in male), *L. sungi* (48.3–52.7 mm in males and 56.7–58.9 mm in females), and *L. zhangyapingi* (45.8–52.5 mm in males), and differs from the smaller *L. aerea* (25.1–28.9 mm in males), *L. alpina* (24.0–26.4 mm in males), *L. applebyi* (19.6–22.3 mm in males), *L. ardens* (21.3–24.7 mm in males), *L. baluensis* (14.9–15.9 mm in males), *L. bidoupensis* (18.5–25.4 mm in males), *L. bijie* (29.0–30.4 mm in males), *L. bondangensis* (17.8 mm in male), *L. brevicrus* (17.1–17.8 mm in males), *L. crocea* (22.2–27.3 mm in males), *L. frthi* (26.4–29.2 mm in males), *L. fuliginosa* (28.2–30.0 mm in males), *L. fusca* (16.3 mm in male), *L. isos* (23.7–27.9 mm in males), *L. itiokai* (15.2–16.7 mm in males), *L. juliandringi* (17.0–17.2 mm in males and 18.9–19.1 mm in females), *L. khasiorum* (24.5–27.3 mm in males), *L. lateralis* (26.9–28.3 mm in males), *L. laui* (24.8–26.7 mm in males), *L. liui* (23.0–28.7 mm in males), *L. macrops* (28.0–29.3 mm in males), *L. maculosa* (24.2–26.6 mm in males), *L. mangshanensis* (22.22–27.76 mm in males), *L. melica* (19.5–22.8 mm in males), *mjobergi* (15.7–19.0 mm in males), *L. natunae* (17.6 mm in male), *L. pallida* (24.5–27.7 mm in males), *L. palmata* (14.4–16.8 mm in males), *L. parva* (15.0–16.9 mm in males), *L. petrops* (23.6–27.6 mm in males), *L. pluvialis* (21.3–22.3 mm in males), *L. purpuraventra* (27.3–29.8 mm in males), *L. puhoatensis* (24.2–28.1 mm in males), *L. purpura* (25.0–27.5 mm in males), *L. rowleyae* (23.4–25.4 mm in males), *L. shangsiensis* (24.9–29.4 mm in males), *L. suiyangensis* (28.7–29.7 mm in males), *L. tadungensis* (23.3–28.2 mm in males), *L. tengchongense* (23.9–26.0 mm in males), *L. tuberosa* (24.4–29.5 mm in males), *L. ventripunctata* (25.5–28.0 mm in males), *L. wuhuangmontis* (25.6–30.0 mm in males), *L. yingjiangensis* (25.7–27.6 mm in males), and *L. yunkaiensis* (25.9–29.3 mm in males).

**Table 4.** Diagnosis characters on morphology of *Leptobrachella chishuiensis* sp. nov. from other congeners.

ID	Species	Male SVL (mm)	Black spots on flanks	Toes webbing	Fringes on toes	Ventral coloration	Dorsal skin texture
1	*Leptobrachella chishuiensis* sp. nov.	30.8–33.4	Yes	Rudimentary	Narrow	White with distinct nebulous greyish speckling on chest and ventrolateral flanks	Shagreened and granular
2	*L. aerea*	25.1–28.9	No	Rudimentary	Wide	Near immaculate creamy white, brown specking on margins	Finely tuberculate
3	*L. alpina*	24.0–26.4	Yes	Rudimentary	Wide in males	Creamy-white with dark spots	Relatively smooth, some with small warts
4	*L. applebyi*	19.6–22.3	Yes	Rudimentary	No	Reddish brown with white speckling	Smooth
5	*L. ardens*	21.3–24.7	Yes	No	No	Reddish brown with white speckling	Smooth- finely shagreened
6	*L. bidoupensis*	18.5–25.4	Yes	Rudimentary	Weak	Reddish brown with white speckling	Smooth
7	*L. bijie*	29.0–30.4	Yes	Rudimentary	Narrow	White with distinct nebulous greyish speckling on chest and ventrolateral flanks	Shagreened and granular
8	*L. botsfordi*	29.1–32.6	No	Rudimentary	Narrow	Reddish brown with white speckling	Shagreened
9	*L. bourreti*	28.0–36.2	Yes	Rudimentary	Weak	Creamy white	Relatively smooth, some with small warts
10	*L. crocea*	22.2–27.3	No	Rudimentary	No	Bright orange	Highly tuberculate
11	*L. eos*	33.1–34.7	No	Rudimentary	Wide	Creamy white	Shagreened
12	*L. firthi*	26.4–29.2	No	Rudimentary	Wide in males	Creamy white	Shagreened with fine tubercles
13	*L. fuliginosa*	28.2–30.0	Yes	Rudimentary	Weak	White with brown dusting	Nearly smooth, few tubercles
14	*L. isos*	23.7–27.9	No	Rudimentary	Wide in males	Creamy white with white dusting on margins	Mostly smooth, females more tuber- culate
15	*L. kalonensis*	25.8–30.6	Yes	No	No	Pale, speckled brown	Smooth
16	*L. khasiorum*	24.5–27.3	Yes	Rudimentary	Wide	Creamy white	Isolated, scattered tubercles
17	*L. lateralis*	26.9–28.3	Yes	Rudimentary	No	Creamy white	Roughly granular
18	*L. laui*	24.8–26.7	Yes	Rudimentary	Wide	Creamy white with dark brown dusting on margins	Round granular tubercles
19	*L. liui*	23.0–28.7	Yes	Rudimentary	Wide	Creamy white with dark brown spots on chest and margins	Round granular tubercles with glandular folds
20	*L. macrops*	28.0–29.3	Yes	Rudimentary	No	Greyish-violet with white speckling	Roughly granular with larger tubercles
21	*L. maculosa*	24.2–26.6	Yes	No	No	Brown, less white speckling	Mostly smooth
22	*L. mangshanensis*	22.22–27.76	Yes	Rudimentary	Weak	White speckles on throat and belly	Nearly smooth
23	*L. maoershanensis*	25.2–30.4	Yes	Rudimentary	Narrow	Creamy white chest and belly with irregular black spots	Longitudinal folds
24	*L. marmorata*	32.3–38.0	Yes	Rudimentary	No	Chest and belly immaculate white	Nearly smooth, scattered with small tubercles of varying sizes
25	*L. melica*	19.5–22.8	Yes	Rudimentary	No	Reddish brown with white speckling	Smooth
26	*L. minima*	25.7–31.4	Yes	Rudimentary	No	Creamy white	Smooth
27	*L. nahangensis*	40.8	Yes	Rudimentary	No	Creamy white with light specking on throat and chest	Smooth
28	*L. namdongensis*	30.9	Yes	Rudimentary	No	Creamy white with brown dusting on margins	Finely tuberculate
29	*L. nokrekensis*	26.0–33.0	Yes	Rudimentary	unknown	White with distinct nebulous greyish speckling on chest and ventrolateral flanks	Tubercles and longitudinal folds
30	*L. nyx*	26.7–32.6	Yes	Rudimentary	No	Creamy white with white with brown margins	Rounded tubercles
31	*L. oshanensis*	26.6–30.7	Yes	No	No	Whitish with no markings or only small, light grey spots	Smooth with few glandular ridges
32	*L. pallida*	24.5–27.7	No	No	No	Reddish brown with white speckling	Tuberculate
33	*L. pelodytoides*	27.5–32.3	Yes	Wide	Narrow	Whitish	Small, smooth warts
34	*L. petrops*	23.6–27.6	No	No	Narrow	Immaculate creamy white	Highly tuberculate
35	*L. pluvialis*	21.3–22.3	Yes	Rudimentary	No	Dirty white with dark brown marbling	Smooth, flattened tubercles on flanks
36	*L. puhoatensis*	24.2–28.1	Yes	Rudimentary	Narrow	Reddish brown with white dusting	Longitudinal skin ridges
37	*L. purpura*	25.0–27.5	Yes	Rudimentary	Wide	Dull white with indistinct grey dusting	Shagreen with small tubercles
38	*L. purpuraventra*	27.3–29.8	Yes	Rudimentary	Narrow	Grey purple with distinct nebulous greyish speckling on chest and ventrolateral flanks	Shagreened and granular
39	*L. pyrrhops*	30.8–34.3	Yes	Rudimentary	No	Reddish brown with white speckling	Slightly shagreened
40	*L. rowleyae*	23.4–25.4	Yes	No	No	Pinkish milk-white to light brown chest and belly with numerous white speckles	Smooth with numerous tiny tubercles
41	*L. sabahmontana*	25–28	Yes	Rudimentary	Narrow	Cream-coloured with dark brown speckling	with tiny tubercles, weakly wrinkled
42	*L. shangsiensis*	24.9–29.4		Rudimentary	Narrow	Ventral surface yellowish creamy-white with marble texture	Smooth
43	*L. sungi*	48.3–52.7	No or small	Wide	Weak	White	Granular
44	*L. suiyangensis*	28.7–29.7	Yes	Rudimentary	Narrow	Yellowish creamy-white with marble texture chest and belly or with irregular light brown speckling	Shagreen with small granules
45	*L. tadungensis*	23.3–28.2	Yes	No	No	Reddish brown with white speckling	Smooth
46	*L. tamdil*	32.3	Yes	Wide	Wide	White	Weakly tuberculate
47	*L. tengchongense*	23.9–26.0	Yes	Rudimentary	Narrow	White with dark brown blotches	Shagreened with small tubercles
48	*L. tuberosa*	24.4–29.5	No	Rudimentary	No	White with small grey spots/streaks	Highly tuberculate
49	*L. ventripunctata*	25.5–28.0	Yes	Rudimentary	No	Chest and belly with dark brown spots	Longitudinal skin ridges
50	*L. wuhuangmontis*	25.6–30.0	Yes	Rudimentary	Narrow	Greyish white mixed by tiny white and black dots	Rough, scattered with dense conical tubercles
51	*L. yingjiangensis*	25.7–27.6	Yes	Rudimentary	Wide	Creamy white with dark brown flecks on chest and margins	Shagreened with small tubercles
52	*L. yunkaiensis*	25.9–29.3	Yes	Rudimentary	Wide	Belly pink with distinct or indistinct speckling	Shagreened with short skin ridges and raised warts
53	*L. zhangyapingi*	45.8–52.5	No	Rudimentary	Wide	Creamy-white with white with brown	Mostly smooth with distinct tubercles

By supra-axillary and ventrolateral glands present, *Leptobrachella chishuiensis* sp. nov. differs from *L. arayai*, *L. dringi*, *L. fritinniens*, *L. gracilis*, *L. hamidi*, *L. heteropus*, *L. kajangensis*, *L. kecil*, *L. marmorata*, *L. melanoleuca*, *L. maura*, *L. picta*, *L. platycephala*, *L. sabahmontana*, and *L. sola* (vs. absent in the latter).

By having black spots on ﬂanks, *Leptobrachella chishuiensis* sp. nov. differs from *L. aerea*, *L. botsfordi*, *L. frthi*, and *L. tuberosa* (vs. lacking in the latter).

By toes with rudimentary webbing, *Leptobrachella chishuiensis* sp. nov. differs from *L. kalonensis* and *L. oshanensis* (vs. lacking webbing on toes in the latter), and differs from *L. pelodytoides* (vs. toes with wide webbing in the latter).

By having shallow lateral fringes on toes, *Leptobrachella chishuiensis* sp. nov. differs from *L. aerea*, *L. frthi*, *L. liui*, and *L. yunkaiensis* (vs. having prominently wide lateral fringes on toes in the latter), and differs from *L. kalonensis*, *L. macrops*, *L. minima*, *L. nyx*, *L. oshanensis*, *L. pyrrhops*, and *L. tuberosa* (vs. lacking lateral fringes on toes in the latter).

By having dorsal surface shagreened and granular, lacking enlarge tubercles or warts, *Leptobrachella chishuiensis* sp. nov. differs from the following species: *L. bourreti* (dorsum smooth with small warts), *L. fuliginosa* (dorsum smooth with fine tubercles), *L. liui* (dorsum with round tubercles), *L. macrops* (dorsum roughly granular with large tubercles), *L. maoershanensis* (dorsum shagreened with tubercles), *L. minima* (dorsum smooth), *L. nyx* (dorsum with round tubercles), *L. pelodytoides* (dorsum with small, smooth warts), *L. tamdil* (dorsum weakly tuberculate, with low, oval tubercles), *L. tuberosa* (dorsum higly tuberculate), *L. yunkaiensis* (dorsum with raised warts), and *L. wuhuangmontis* (dorsum rough with conical tubercles).

By the finger II < I, *Leptobrachella chishuiensis* sp. nov. differs from *L. tamdil* (vs. II > I in the latter).

By head length slightly longer than wide, *Leptobrachella chishuiensis* sp. nov. differs from *L. namdongensis* (vs. head wider than long in the latter).

Six *Leptobrachella* species were reported to be distributed in Guizhou Province, China, they are: *L. liui*, *L. oshanensis*, *L. purpuraventra*, *L. bijie*, *L. ventripunctata*, and *L. suiyangensis* (Fei et al. 2012; Li et al. 2016; Wang et al. 2019; Luo et al. 2020). We make a comparative note between them and the new species as follows. *Leptobrachella chishuiensis* sp. nov. differs from *L. liui* by having shallow lateral fringes on toes (vs. wide lateral fringes on the toes in the latter), dorsal surface shagreened with small granules, lacking enlarge tubercles or warts (vs. dorsum with round tubercles in the latter); from *L. oshanensis* by having rudimentary webbing on the toes (vs. lack webbing on the toes in the latter), having shallow lateral fringes on toes (vs. lacking lateral fringes on the toes in the latter), from *L. suiyangensis* by heels overlapping when thighs are positioned at right angles to the body (vs. just meeting in the latter), tibia-tarsal articulation reaches tympanum or tympanum to eye (vs. reaches to the anterior corner of eye in the latter); from *L. ventripunctata* by bigger body size (SVL 30.8–33.4 mm in adult males vs. SVL 25.5–28.0 mm in males in the latter), chest and belly without large dark brown spots (vs. with large dark brown spots in the latter).

*Leptobrachella chishuiensis* sp. nov. is genetically closer to *L. bijie* and *L. purpuraventra*. The new species differs from *L. bijie* by the following characters: larger body size (SVL 30.8–33.4 mm in males vs. SVL 29.0–30.4 mm in males in the latter), internasal distance longer than interorbital distance (vs. equal to interorbital distance in the latter), heels overlapping (vs. just meeting in the latter), tibia-tarsal articulation reaches the tympanum or tympanum to eye (vs. reaching the region between middle of eye to anterior corner of eye in the latter), one call contains 1–4 notes (vs. 2 notes in each call in the latter), having shorter call interval (60 ± 21, *N* = 31 in the new species vs. 101.9 ± 6.4, *N* = 33 in the latter), having significantly higher value of SVL in males, and having significantly higher value of HDL, HDW, SL, IND, IOD, TEY, TL and FL to SVL in males (all P-values < 0.05; Table 5).

*Leptobrachella chishuiensis* sp. nov. differs from *L. purpuraventra* by larger body size (SVL 30.8–33.4 mm in seven adult males vs. SVL 27.3–29.8 mm in eleven adult males in the latter), tibia-tarsal articulation reaches the tympanum or tympanum to eye (vs. reaching the middle of eye in the latter), the call contains 1–4 notes (vs. 2 notes in each call in the latter), having longer call duration (200 ± 67, *N* = 32 vs. 192.2 ± 13.0 as the longest call duration in *L. purpuraventra*), shorter call interval (60 ± 21, *N* = 31 vs. 90.8 ± 5.6, *N* = 20 as the shortest call interval in *L. bijie*), having significantly higher value of SVL in males, and having significantly higher value of SVL, HDL,HDW, SL, IOD, ED, TYD, LAL, TL and FL to SVL in males (all P-values < 0.05; Table 5).

**Table 5.** Morphometric comparisons between *Leptobrachella chishuiensis* sp. nov and its relatives. Units in mm. See abbreviations for morphometric characters in Materials and methods section.

	***Leptobrachella chishuiensis* sp. nov.**			***L. bijie***		***L. purpuraventra***		***P*-value from Mann-Whitney *U* test**	
	**Male (*N* = 7)**		**Female (*N* = 1)**	**Male (*N* = 8)**		**Male (*N* = 11)**			
	**Range**	**Mean ± SD**		**Range**	**Mean ± SD**	**Range**	**Mean ± SD**	***L. chishuiensis* vs. *L. bijie***	***L. chishuiensis* vs. *L. purpuraventra***
SVL	30.8–33.4	32.1 ± 1.0	34.2	29.0–30.4	29.7 ± 0.6	27.3–29.8	28.9 ± 0.8	0.001	0.000
HDL	11.1–12.3	11.8 ± 0.4	12.7	10.0–10.6	10.2 ± 0.2	9.6–10.3	9.9 ± 0.3	0.021	0.013
HDW	10.6–11.9	11.4 ± 0.5	12.0	9.5–10.2	9.8 ± 0.3	9.3–9.8	9.6 ± 0.2	0.001	0.001
SL	4.8–5.8	5.2 ± 0.3	5.3	4.0–4.7	4.2 ± 0.2	3.5–4.1	3.8 ± 0.2	0.002	0.000
IND	3.5–3.8	3.7 ± 0.1	3.4	2.8–3.4	3.1 ± 0.2	2.7–3.5	3.1 ± 0.2	0.003	0.094
IOD	2.7–3.1	3.0 ± 0.2	2.7	2.8–3.4	3.1 ± 0.2	2.6–3.2	2.9 ± 0.2	0.008	0.016
UEW	3.0–3.3	3.2 ± 0.1	3.0	/	/	/	/	/	/
ED	4.0–5.0	4.4 ± 0.4	4.4	3.6–4.1	3.8 ± 0.2	3.1–3.6	3.4 ± 0.2	0.064	0.001
TYD	2.0–2.6	2.3 ± 0.2	2.4	1.9–2.2	2.0 ± 0.1	1.7–1.9	1.8 ± 0.1	0.247	0.000
TEY	1.2–1.6	1.4±0.2	1.2	0.9–1.1	1.0 ± 0.1	1.1–1.3	1.2 ± 0.1	0.002	0.751
LAL	14.7–17.0	15.6 ± 0.8	16.3	14.0–14.8	14.3 ± 0.3	12.6–14.0	13.3 ± 0.4	0.643	0.016
LW	2.6–3.2	3.0 ± 0.2	3.3	/	/	/	/	/	/
ML	7.9–8.8	8.2± 0.39	8.7	7.4–8.3	7.8 ± 0.3	7.0–7.7	7.4 ± 0.2	0.247	0.964
FIL	3.0–3.8	3.4 ± 0.3	3.4	/	/	/	/	/	/
FIIL	2.8–3.4	3.0 ± 0.2	3.0	/	/	/	/	/	/
FIIIL	4.9–5.5	5.1 ± 0.2	5.6	/	/	/	/	/	/
FIVL	2.9–3.5	3.2 ± 0.2	3.3	/	/	/	/	/	/
HLL	43.3–49.7	49.7 ± 2.7	49.4	43.0–45.5	43.7 ± 0.8	39.0–44.6	41.4 ± 2.2	0.487	0.113
THL	13.7–17.1	15.1 ± 1.2	15.3	/	/	/	/	/	/
TW	3.3–4.3	3.8 ± 0.4	4.2	/	/	/	/	/	/
TL	14.9–16.8	15.6 ± 0.6	16.0	13.5–14.4	13. ± 0.3	12.5–14.0	13.1 ± 0.5	0.005	0.001
TFL	20.9–22.3	21.7 ± 0.6	22.2	/	/	/	/	/	/
FL	14.4–15.9	15.1 ± 0.5	16.3	13.0–13.8	13.3 ± 0.2	12.1–13.2	12.6 ± 0.4	0.004	0.000

#### Ecology.

**Figure 8.**
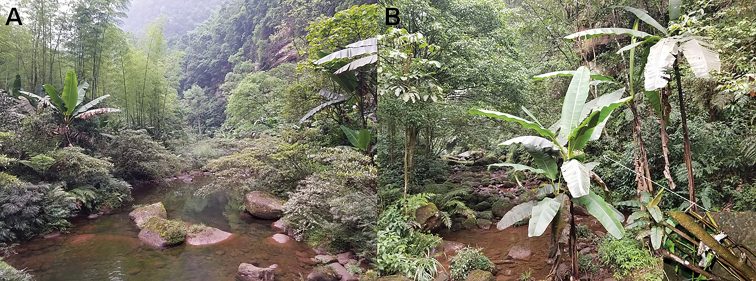
Habitats of *Leptobrachella chishuiensis* sp. nov. in the type locality Chuishui National Nature Reserve, Chishui City, Guizhou Province, China **A** landscape of montane forests in the type locality **B** a mountain stream in the type locality (*insert* holotype CIBCS20190518047 in life in the field).

*Leptobrachella chishuiensis* sp. nov. is known from the type locality, Chishui National Nature Reserve (28.383333–28.45N, 105.05–109.75E), Chishui City, Guizhou Province, China at elevations between 270–604 m a. s. l. This new species is found in bamboo forest nearby the streams (Fig. 8), and four sympatric amphibian species, i.e. *Megophrys omeimontis*, *Odorrana margaratae* (Liu, 1950), *Zhangixalus omeimontis* (Stejneger, 1924), and *Rana omeimontis* Ye & Fei, 1993 were found nearby.

#### Etymology.

This specific name *chishuiensis* refers to the distribution of this species, Chishui City, Guizhou Province, China. We propose the common English name “Chishui leaf litter toads” (English) and its Chinese as “Chi Shui Zhang Tu Chan (赤水掌突蟾)”.

## Discussion

The Asian leaf litter toads of *Leptobrachella* have low vagility and are in exclusive association with montane forests, and their populations are often highly structured. Underestimation of species diversity occurs in the genus, which suggests a high degree of localized diversification and micro-endemism (Fei et al. 2012; Chen et al. 2018). Many cryptic species were proposed by molecular analyses in areas where surveys are weak (Chen et al. 2018), but in Guizhou Province the investigation into the genus was poor although this area was likely to be an important transition zone for many clades or lineages (Chen et al. 2018). Additionally, in Guizhou Province, many new amphibian species has been described in recent years (Zhang et al. 2017; Li et al. 2018a, b; Li et al. 2019a, b; Lyu et al. 2019; Wang et al. 2019; Wei et al. 2020), including two species of *Leptobrachella*, indicating the underestimated species diversity of amphibians in this region. To date, in Guizhou Province, seven *Leptobrachella* species were recorded, i.e., *Leptobrachella chishuiensis* sp. nov., *L. liui*, *L. oshanensisL. purpuraventra*, *L. bijie*, *L. ventripunctata*, and *L. suiyangensis* (Fei et al. 2012; Li et al. 2016; Wang et al. 2019; Luo et al. 2020). It is expected that in this area, the species diversity of *Leptobrachella* may be underestimated, and more investigation should be conducted for detecting richness of the toad species.

The new species is found along clear water rocky streams from Chishui County, Guizhou Province, China, and little is known about the population status of the new species. Thus, further research on the true distribution, population size and trends, and conservation actions are required.

## Supplementary Material

XML Treatment for Leptobrachella chishuiensis

## Supplementary materials

Supplementary material 1 Table S1. Uncorrected *p*-distance between *Leptobrachella* species on the 16S rRNA gene Data type genetic distance Explanation note Mean value of genetic distance is given in the lower half of the table. https://binary.pensoft.net/file/424105This dataset is made available under the Open Database License (http://opendatacommons.org/licenses/odbl/1.0/). The Open Database License (ODbL) is a license agreement intended to allow users to freely share, modify, and use this Dataset while maintaining this same freedom for others, provided that the original source and author(s) are credited.Shi-Ze Li, Jing Liu, Gang Wei, Bin Wang
